# The association of anthropometry indices with gout in Taiwanese men

**DOI:** 10.1186/1472-6823-13-30

**Published:** 2013-08-15

**Authors:** Wen-Yu Lin, Chia-Chi Lung, Ting-Sung Liu, Zhi-Hong Jian, Pei-Chieh Ko, Jing-Yang Huang, Chien-Chang Ho, Shih-Chang Chen, Yi-Chen Chiang, Yung-Po Liaw

**Affiliations:** 1 Jen-Ai Hospital, Taichung City, 41265, Taiwan; 2 Department of Public Health and Institute of Public Health, Chung Shan Medical University, Taichung City 40201, Taiwan; 3 Department of Family and Community Medicine, Chung Shan Medical University Hospital, Taichung City 40201, Taiwan; 4 Department of Health and Leisure Management, Yuanpei University, Hsinchu City 300, Taiwan; 5 Department of Leisure Industry and Health Promotion, National Ilan University, Yilan City 260, Taiwan

**Keywords:** Gout, Anthropometry, Waist to height ratio

## Abstract

**Background:**

To examine the association of anthropometry indices with gout and to compare the performance of indices to predict gout in Taiwanese men.

**Methods:**

There were 1443 male subjects aged more than 20 years who participated in the Nutrition and Health Survey in Taiwan (NAHSIT, 1993–1996). Anthropometric evaluation consisted of weight, height, hip and waist circumference (WC) with later body mass index (BMI), waist to height (WHtR) and waist to hip (WHR) estimations. We conducted 4 logistic models to determine the relationships between anthropometric indices and gout. Receiver operating characteristic (ROC) curve were used to compare the predictive performance and to identify the optimal cut-off points, sensitivity and specificity of these indices for gout in men.

**Results:**

After controlling for other covariables, the adjusted odds ratios for the mid and top tertiles of WHtR were 2.55 (95% CI: 1.16, 5.59) and 3.01 (95% CI: 1.13, 7.99), respectively, but no linear association was found for BMI, WHR and WC. In ROC curve, the greatest area under curve was 0.684 for WHtR and the cut-off point of WHtR was 0.57.

**Conclusions:**

WHtR had a significant linear association with gout in Taiwanese men and was superior to BMI, WHR and WC.

## Background

The prevalence of gout has increased in recent years [1]. Twenty-six percent of adult males (≥19 years) and 22% of older males (≥45 years) either had hyperuricemia or were taking medication for it in Nutrition and Health Survey in Taiwan (NAHSIT, 1993–1996) [2]. The data from the Taiwan National Health Insurance Research Database showed that the prevalence of gout was 4.26% and male to female ratio was 3.2:1 [3]. Subjects with high serum uric acid levels are at higher risk for all-cause and cardiovascular mortality [4]. Gout increases the risk of cardiovascular disease, even in young people and those without cardiovascular risk factors [5]. Gout also increases mortality risk in both genders, especially in kidney diseases, endocrine and metabolic diseases, and cardiovascular diseases [6].

Overweight and obesity are epidemic and obese people often have hyperuricemia. Increased serum uric acid [7] and gout occurrence [8] have been known to be closely associated with an increase in visceral fat accumulation. The significance of visceral fat obesity has been found to be associated with chronic metabolic disorders [9]. Insulin resistance plays a key role in the connection between hyperuricemia and metabolic syndrome [10,11]. It is known that body mass index (BMI), triglycerides, hypertension, inflammation and insulin resistance increase the uric acid concentration [12-14]. High plasma urate concentration is associated with articular deposits of urate crystals that cause gout [15]. The predisposing factors such as alcohol consumption, diuretics use, and excess weight gain increase risks of gout attack among patients with hyperuricemia [16]. Gout has also been linked with metabolic syndrome and diabetes [17]. Obesity is positively associated with gout, whereas body weight loss is protective [18].

Thus, on the basis of these evidences, it is important to find an easy, practical screening index for predicting gout in the general male Taiwanese population. However, the evidence about the correlation of BMI, waist circumference (WC), waist to hip ratio (WHR), or waist to height ratio (WHtR) with gout in the Taiwanese population is scant. We tried to assume that other anthropometric index rather than BMI could more accurately predict gout. Therefore, the aim of this study was to ascertain which anthropometric index can be better at predicting gout in the Taiwanese men.

## Methods

### Study participants

The data were obtained from the Nutrition and Health Survey in Taiwan that was performed between 1993 and 1996 (NAHSIT, 1993–1996). NAHSIT was a nationwide survey for the nutrition and health status of Taiwanese. All instruments of the NAHSIT 1993–1996 were evaluated for validity and reliability in 3 pilot studies conducted from January to June in 1993 [19]. All subjects were gave written informed consent for participation before enrollment in the study. The database consists of de-identified secondary data and is released for purposes of public researches.

The details of the design and the operational techniques about the survey have been described previously [20]. Briefly, data were drawn from 7 geographical district samples according to their unique dietary patterns of the residents, ancestral origins, geographic location, and degree of urbanization (Hakka people areas, mountainous areas, the East coast area, the Peng-Hu islands, metropolitan cities, urbanization Class I townships, and urbanization Class II townships).

### Data collection

After the strata were classified, 3 townships or city districts were selected within each stratum by selection probability proportional to its population size (PPS). Three communities were selected in each township or city district by PPS method. People in each communities were further divided into 7 age groups (4–6, 7–12, 13–15, 16–18, 19–44, 45–64, and ≧ 65 years) and 2 sex groups. Eight or 16 individuals (depending on the available age groups) were sampled in each age group by sex group. A pseudo-Latin square design was used to allocate survey time to balance the effects of season and year in this subtropical land. Demographic and clinical characteristics, and physical examinations were recorded and documented during face-to-face interviews with structured questionnaires. Finally, there were 6464 subjects completed the physical examination and 5843 adequate fasting blood samples. The present study enrolled 1443 Taiwanese males aged ≧20 years with complete questionnaires (sex, age, area, alcohol consumption, and medical history), physical examinations and results of fasting blood samples.

### Definitions

Hyperuricemia was defined as serum uric acid ≧ 7.0 mg/dl for males [21]. Taiwan definitions adopted BMI 24 kg/m^2^ as the cutpoint for overweight and 27 kg/m^2^ for obesity [22]. The definitions of metabolic syndrome in males are WC ≥90 cm and any two of the following: (1) high triglyceride ≥150 mg/dL, (2) low HDL-cholesterol <40 mg, (3) high blood pressure ≥130/85 mg or on medication, (4) raised fasting plasma glucose ≧100 mg/dl or diabetes [23]. We defined gout as reporting gout diagnosed by a physician from questionnaires.

The self-reported alcohol consumption was categorized into low, moderate and high. Anthropometric variables included height, weight, WC and hip circumference. Hip circumference was measured at the level of the greater trochanter. WC was measured at the mid-level between the iliac crest and the last rib. BMI was calculated as weight (kilogram) divided by height (meter) squared, WHR was calculated as WC (centimeter) divided by HC (centimeter), and WHtR was calculated as WC (centimeter) divided by height (centimeter).

### Statistical analyses

All analyses were carried out by using the SAS ver. 9.1 software package (SAS Institute, Cary, NC, USA). Comparisons between gout and non-gout subjects’ demographic data, laboratory measurements, and anthropometric indices were made using Student’s *t* test and Chi-square tests. Adjusted odds ratios (ORs) with 95% confidence intervals (CIs) for gout were calculated from the multiple logistic regression analysis according to obesity (BMI ≥ 27 kg/m^2^ and top tertile of WC, WHR, and WHtR) and the tertiles of the indices. In the current study, the ROC curve analyses were performed by calculating the true positive rate (sensitivity) against the false-positive rate (1 − specificity) to assess and measure the predictive power of each anthropometric index in the association with gout based on the area under the curve (AUC). A *p* value <0.05 was considered statistically significant.

## Results

The basic characteristics of the 104 gout and 1339 non-gout male participants aged ≧ 20 years are shown in Table 1. The age of gout group was older than non-gout Taiwanese. The highest prevalence of gout was found in the mountainous areas. The prevalence of high anthropometric indices, metabolic syndrome, dyslipidemia, high blood pressure and hyperuricemia were significantly higher in gout group than that in non-gout group. There were no significant differences in smoking, alcohol drinking and renal disease between the two groups.

**Table 1:** Demographic, clinical, laboratory and anthropometric characteristics of subjects with and without gout

**Variables**	**Gout (n = 104)**	**Non gout (n = 1339)**	**p-value**
Age (year)	55.1 ± 13.7	51.1 ± 15.7	0.01
Area (%)			<0.01
Hakka area	13.5	14.3	
Mountainous area	37.5	13.7	
East Coast area	9.6	15.3	
Penghu islands	10.6	14.0	
Metropolitan cities	9.6	14.9	
Provincial class I townships	10.6	13.1	
Provincial class II townships	8.7	14.7	
Lifestyle factors			
Smoking (%)	68.6	70.9	0.63
Alcohol drinking (%)			
Low	17.7	19.7	0.80
Moderate	54.9	51.6	
High	27.5	28.7	
Hypertension medication (%)	21.2	8.2	<0.01
Anthropometric indices			
BMI (kg/m^2^)	25.2 ± 2.9	23.5 ± 3.4	<0.01
WC (cm)	86.3 ± 7.6	81.3 ± 9.8	<0.01
WHR	0.90 ± 0.05	0.87 ± 0.07	<0.01
WHtR	0.53 ± 0.04	0.49 ± 0.06	<0.01
Renal disease (%)	4.9	2.5	0.15
Metabolic syndrome (%)	41.4	16.9	<0.01
Triglyceride ≧ 150 mg/dl (%)	45.2	24.1	<0.01
HDL <40 mg/dl (%)	37.5	28.5	0.05
High blood pressure (%)*	71.2	53.6	0.01
High fasting glucose (%)^#^	15.4	12.0	0.31
Uric acid ≧ 7.0 mg/dl (%)	64.4	40.3	<0.01

Data are expressed as mean ± standard deviation, unless otherwise indicated.

Abbreviations: *BMI* Body mass index, *HDL* High density lipoprotein, *WC* Waist circumference, *WHR* Waist to hip ratio, *WHtR* Waist to height ratio.

*Blood pressure ≧ 130/85 mmHg or participants with medication control.

^#^ Fasting glucose ≧ 100 mg/dl or participants with medication control.

In Table 2, BMI ≥27 kg/m^2^ and the top tertile of WC, WHR and WHtR were considered to be obese. It shows the ORs for gout by BMI ≥ 27 kg/m^2^ and top tertile of WC, WHR, and WHtR after adjusting for age, smoking, areas and hypertension medications. Hyperuricemia and metabolic syndrome were significantly associated with gout in all models. The adjusted OR for gout was significantly increased in the top tertile of WC (OR, 1.70; 95% CI, 1.04-2.79), WHR (OR, 1.77; 95% CI, 1.07-2.92), and WHtR (OR, 2.28; 95% CI, 1.35-3.86). A non-significant result was found for BMI (≧27 kg/m^2^) (OR, 1.59; 95% CI, 0.98-2.57) in Model 4.

**Table 2:** The ORs for gout in relation to obesity definitions after adjustment for potential confounders

**Variables**		**Model 1**		**Model 2**		**Model 3**		**Model 4**	
		**OR**	**95% CI**	**OR**	**95% CI**	**OR**	**95% CI**	**OR**	**95% CI**
Hyperuricemia									
	No	1.00		1.00		1.00		1.00	
	Yes	1.90	(1.21, 3.00)	1.92	(1.22, 3.02)	1.87	(1.19, 2.96)	1.92	(1.22, 3.02)
Renal disease									
	No	1.00		1.00		1.00		1.00	
	Yes	1.32	(0.46, 3.77)	1.36	(0.48, 3.85)	1.31	(0.46, 3.74)	1.22	(0.43, 3.50)
Alcohol drinking									
	Low	1.00		1.00		1.00		1.00	
	Moderate	1.05	(0.58, 1.90)	1.07	(0.59, 1.92)	1.03	(0.57, 1.86)	1.08	(0.60, 1.95)
	High	0.83	(0.42, 1.65)	0.81	(0.41, 1.61)	0.81	(0.49, 1.62)	0.89	(0.45, 1.75)
Metabolic syndrome									
	No	1.00		1.00		1.00		1.00	
	Yes	1.85	(1.14, 3.02)	1.84	(1.13, 3.00)	1.71	(1.06, 2.77)	1.88	(1.15, 3.07)
Obesity*									
WC (cm)	≧ 88.1	1.70	(1.04, 2.79)						
WHR	≧ 0.91			1.77	(1.07, 292)				
WHtR	≧ 0.53					2.28	(1.35, 3.86)		
BMI (kg/m^2^)	≧ 27							1.59	(0.98, 2.57)

Adjustment for age, smoking, areas and hypertension medication in all models.

Abbreviations: *BMI* Body mass index, *WC* Waist circumference, *WHR* Waist to hip ratio, *WHtR* Waist to height ratio.

*Obesity is defined as BMI ≥ 27 kg/m^2^ and top tertile of WC,WHR, and WHtR.

Four multivariable models of gout consisting of variables for renal disease, alcohol drinking, metabolic syndrome, hyperuricemia and anthropometric indices after adjustment for age, smoking, areas and hypertension medication are shown in Table 3. The OR for gout was significantly increased in the mid and top tertiles of WC in Model 1 (p trend = 0.03). We simultaneously adjusted WC into the Model 2, 3, and 4. After the adjustments, the ORs for gout increased significantly for WHtR, but not for BMI and WHR. The adjusted odds ratios for the mid and top tertiles of WHtR were 2.55 (95% CI: 1.16, 5.59) and 3.01 (95% CI: 1.13, 7.99) in Model 3, respectively. The obesity index, WHtR, seemed to have more stable effects on gout than did WC.

**Table 3:** The ORs for gout in relation to tertiles of each anthropometric index after adjustment for potential confounders

**Variables**		**Model 1**		**Model 2**		**Model 3**		**Model 4**	
		**OR**	**95% CI**	**OR**	**95% CI**	**OR**	**95% CI**	**OR**	**95% CI**
Renal disease	No	1.00		1.00		1.00		1.00	
	Yes	1.47	(0.51, 4.19)	1.55	(0.55, 4.39)	1.45	(0.51, 4.13)	1.43	(0.50, 4.10)
Alcohol drinking	Low	1.00		1.00		1.00		1.00	
	Moderate	1.08	(0.60, 1.96)	1.09	(0.60, 1.97)	1.08	(0.59, 1.98)	1.10	(0.61, 2.00)
	High	0.85	(0.42, 1.70)	0.82	(0.41, 1.65)	0.85	(0.42, 1.72)	0.88	(0.44, 1.77)
Metabolic disorders*									
Hypertriglyceridemia	No	1.00		1.00		1.00		1.00	
	Yes	1.70	(1.06, 2.72)	1.65	(1.03, 2.64)	1.64	(1.02, 2.63)	1.70	(1.06, 2.72)
Low HDL	No	1.00		1.00		1.00		1.00	
	Yes	1.13	(0.72, 1.79)	1.11	(0.70, 1.75)	1.10	(0.70, 1.75)	1.13	(0.72, 1.79)
High blood pressure	No	1.00		1.00		1.00		1.00	
	Yes	1.14	(0.69, 1.88)	1.14	(0.69, 1.87)	1.12	(0.68, 1.85)	1.11	(0.68, 1.84)
High fasting glucose	No	1.00		1.00		1.00		1.00	
	Yes	0.78	(0.42, 1.45)	0.75	(0.40, 1.40)	0.78	(0.41, 1.46)	0.76	(0.41, 1.43)
Hyperuricemia	No	1.00		1.00		1.00		1.00	
	Yes	1.78	(1.12, 2.85)	1.77	(1.11, 2.83)	1.78	(1.12, 2.85)	1.77	(1.11, 2.82)
WC (cm)	<81.7	1.00		1.00		1.00		1.00	
	81.7-88.1	1.81	(1.04, 3.14)	1.47	(0.78, 2.75)	0.91	(0.42, 1.98)	1.52	(0.81, 2.86)
	≧ 88.1	1.83	(1.06, 3.18)	1.38	(0.68, 2.81)	0.78	(0.31, 1.95)	1.35	(0.61, 2.99)
	*P* trend	0.03		0.41		0.61		0.38	
WHR	<0.87			1.00					
	0.87-0.91			1.53	(0.81, 2.88)				
	≧ 0.91			1.57	(0.75, 3.26)				
	*P* trend			0.21					
WHtR	<0.49					1.00			
	0.49-0.53					2.55	(1.16, 5.59)		
	≧ 0.53					3.01	(1.13, 7.99)		
	*P* trend					0.03			
BMI (kg/m^2^)	<24							1.00	
	24-27							1.42	(0.76, 2.65)
	≧ 27							1.50	(0.64, 3.51)
	*P* trend							0.42	

Adjustment for age, smoking, areas, and hypertension medication in all models.

Abbreviations: *BMI* Body mass index, *HDL* High density lipoprotein, *WC* Waist circumference, *WHR* Waist to hip ratio, *WHtR* Waist to height ratio.

*Hypertriglyceridemia: triglyceride ≧ 150 mg/dl; low HDL: HDL < 40 mg/dl; high blood pressure: blood pressure ≧ 130/85 mmHg or hypertension medication; high fasting glucose: glucose ≧ 100 mg/dl or diabetic medication; hyperuricemia: uric acid ≧ 7.0 mg/dl.

Table 4 shows the AUC values of WHtR, BMI, WHR, and WC by using ROC analysis to detect the predictive power of all anthropometric indices for gout. Of the 4 anthropometric indices, WHtR was found to have the largest areas under the ROC curve (0.684). The best combination of sensitivity and specificity for gout was 0.67 and 0.67, respectively. The optimal cut-off values for gout showed WHtR of 0.57.

**Table 4:** Estimated area under curve (AUC) of the indices in predicting gout in men

**Index**	**Estimated AUC**	**95% CI**	**Optimal cut-point**	**Sensitivity**	**Specificity**
WHtR	0.684	(0.639, 0.729)	0.57	0.67	0.67
BMI	0.659	(0.610, 0.708)	27.12	0.72	0.60
WHR	0.657	(0.608, 0.706)	0.89	0.71	0.62
WC	0.655	(0.607, 0.703)	94	0.69	0.64

Abbreviations: *BMI* Body mass index, *WC* Waist circumference, *WHR* Waist to hip ratio, *WHtR* Waist to height ratio.

## Discussion

The proxy measures of visceral fat obesity, principally, WC, WHR or WHtR are used as a surrogate of body fat centralization and have been use for cardiovascular risk evaluation because of their association with cardiometabolic parameters and outcome [9,24,25]. In 2001 Chang et al. [2] reported a nationwide survey from Taiwan that showed strong associations of serum uric acid concentrations with mountainous area, age and BMI in men. A cross-sectional study of 2023 military personnel in Taiwan showed positive associations of serum uric acid with BMI, systolic and diastolic blood pressure, and dyslipidemia [26]. Villegas et al. investigated hyperuricemia in a population consisting middle-aged, urban Chinese men and identified graded positive associations of BMI, WHR, WC, and weight gain [27]. A nationwide study in Taiwan demonstrated that WC has the strongest correlation with hyperuricemia for both men and women than BMI and WHR [28].

In a community based epidemiological study in Taiwan, the risk factors for gout among either the general population or subjects with hyperuricemia were serum uric acid level, alcohol drinking and central obesity [21]. Hypertriglyceridemia in men may potentiate the effect of serum uric acid to increase the risk of gout development [29]. In a large prospective cohort of men, higher BMI and weight gain are strong risk factors for gout in men, while weight reduction is protective [18].

WHtR is a more practical anthropometric index to find higher metabolic risks in normal and overweight Japanese men and women than WC and BMI [30]. A hospital based case–control study of 92 gout and 92 non-gout men showed that WHtR had a significant linear effect on gout risk, independent of BMI [31]. In our study, altered WC increased the chances of gout, but this fact occurred only in the first adjustment of the model in Table 3. When the BMI, WHR and WHtR separately were added, the effect of WC was lost. After adjustment by WC, the OR of WHtR remained significant. From our multivariable models, it suggested that the index of central obesity, especially WHtR, was better index for identifying the risk for gout in this population than were WC and WHR.

One of the strengths of our study was the use of a computerized database, which was population-based and highly representative. A number of important limitations warrant discussion. First, NAHSIT was a cross-sectional study and susceptible to unmeasured confounders and reverse causation. Second, as a result of modernization and life style changes globally, the anthropometric variables of the studied population may have changed. The results from data collected between 1993 and 1996 could be established as a reference or model to which future studies could compare. Third, the diagnosis of gout is based on self reported diagnosis by a physician that might be biased. Although gold standard diagnosis of gout is based upon aspiration of joint fluid for the identification of urate crystals [32], it is impractical for epidemiologic research. Gout is often defined by self-report in epidemiologic studies. Previous population-based cohort study suggested that the self-report of physician diagnosed gout has good reliability and sensitivity and is appropriate for epidemiologic studies [33].

## Conclusion

We had confirmed the hypothesis that WHtR rather than BMI could more accurately predict gout. Among the 4 anthropometric indices used in the present study, although higher values of WC, WHR, and WHtR were associated with gout, WHtR appears to be an independent and better anthropometric index for identifying individuals with gout.

## Abbreviations

AUC: Area under the curve; BMI: Body mass index; CI: Confidence intervals; NAHSIT: Nutrition and Health Survey in Taiwan; OR: Odds ratio; PPS: Probability proportional to population size; ROC: Receiver operating characteristic; WC: Waist circumference; WHtR: Waist to height; WHR: Waist to hip.

## Competing interests

All the authors declare that they have no competing interests.

## Authors’ contributions

YPL designed the study, provided conceptual input and contributed to the final manuscript. TSL, PCK and JYH extracted and analyzed data. WYL, ZHJ and CCL wrote the draft. SCC, YCC and CCH provided conceptual input and contributed to the final manuscript. All authors read and provided feedback on the draft versions of the article. All the authors have read and approved the final version.

## Pre-publication history

The pre-publication history for this paper can be accessed here:

http://www.biomedcentral.com/1472-6823/13/30/prepub

## References

[B1] Chuang SY Lee SC Hsieh YT Pan WH Trends in hyperuricemia and gout prevalence: nutrition and health survey in Taiwan from 1993–1996 to 2005–2008 Asia Pac J Clin Nutr 2011 20 2 301 308 21669599

[B2] Chang HY Pan WH Yeh WT Tsai KS Hyperuricemia and gout in Taiwan: results from the nutritional and health survey in Taiwan (1993–96) J Rheumatol 2001 28 7 1640 1646 11469473

[B3] Kok VC Horng JT Lin HL Chen YC Chen YJ Cheng KF Gout and subsequent increased risk of cardiovascular mortality in non-diabetics aged 50 and above: a population-based cohort study in Taiwan BMC Cardiovasc Disord 2012 12 108 10.1186/1471-2261-12-108 23170782 PMC3556493

[B4] Kuo CF See LC Yu KH Chou IJ Chiou MJ Luo SF Significance of serum uric acid levels on the risk of all-cause and cardiovascular mortality Rheumatology 2013 52 1 127 134 10.1093/rheumatology/kes223 22923756

[B5] Kuo CF Yu KH See LC Chou IJ Ko YS Chang HC Risk of myocardial infarction among patients with gout: a nationwide population-based study Rheumatology 2013 52 1 111 117 10.1093/rheumatology/kes169 22787006

[B6] Kuo CF Yu KH See LC Chou IJ Tseng WY Chang HC Elevated risk of mortality among gout patients: a comparison with the national population in Taiwan Joint Bone Spine 2011 78 6 577 580 10.1016/j.jbspin.2011.01.007 21388850

[B7] Tamba S Nishizawa H Funahashi T Okauchi Y Ogawa T Noguchi M Relationship between the serum uric acid level, visceral fat accumulation and serum adiponectin concentration in Japanese men Intern Med 2008 47 13 1175 1180 10.2169/internalmedicine.47.0603 18591837

[B8] Takahashi S Yamamoto T Tsutsumi Z Moriwaki Y Hada T Increased visceral fat accumulation in patients with primary gout Adv Exp Med Biol 2000 486 131 134 11783469 10.1007/0-306-46843-3_26

[B9] Janssen I Katzmarzyk PT Ross R Waist circumference and not body mass index explains obesity-related health risk Am J Clin Nutr 2004 79 3 379 384 14985210 10.1093/ajcn/79.3.379

[B10] Li C Hsieh MC Chang SJ Metabolic syndrome, diabetes, and hyperuricemia Curr Opin Rheumatol 2013 25 2 210 216 10.1097/BOR.0b013e32835d951e 23370374

[B11] Chou P Lin KC Lin HY Tsai ST Gender differences in the relationships of serum uric acid with fasting serum insulin and plasma glucose in patients without diabetes J Rheumatol 2001 28 3 571 576 11296961

[B12] de Oliveira EP Moreto F Silveira LV Burini RC Dietary, anthropometric, and biochemical determinants of uric acid in free-living adults Nutr J 2013 12 11 10.1186/1475-2891-12-11 23311699 PMC3573899

[B13] Perticone F Sciacqua A Perticone M Arturi F Scarpino PE Quero M Serum uric acid and 1-h postload glucose in essential hypertension Diabetes care 2012 35 1 153 157 10.2337/dc11-1727 22011411 PMC3241313

[B14] So A Thorens B Uric acid transport and disease J Clin Invest 2010 120 6 1791 1799 10.1172/JCI42344 20516647 PMC2877959

[B15] Roddy E Doherty M Epidemiology of gout Arthritis Res Ther 2010 12 6 223 10.1186/ar3199 21205285 PMC3046529

[B16] Lin KC Lin HY Chou P The interaction between uric acid level and other risk factors on the development of gout among asymptomatic hyperuricemic men in a prospective study J Rheumatol 2000 27 6 1501 1505 10852278

[B17] Chen SY Chen CL Shen ML Manifestations of metabolic syndrome associated with male gout in different age strata Clin Rheumatol 2007 26 9 1453 1457 10.1007/s10067-006-0527-4 17256101

[B18] Choi HK Atkinson K Karlson EW Curhan G Obesity, weight change, hypertension, diuretic use, and risk of gout in men: the health professionals follow-up study Arch Intern Med 2005 165 7 742 748 10.1001/archinte.165.7.742 15824292

[B19] Pan WH Kao MD Tzeng MS Yen LL Hung YT Li LA Nutrition and health survey in Taiwan (NAHSIT) 1993–1996: design, contents, and operations Nutr Sci J 1999 24 1 1 10

[B20] Pan WH Chang HY Yeh WT Hsiao SY Hung YT Prevalence, awareness, treatment and control of hypertension in Taiwan: results of Nutrition and Health Survey in Taiwan (NAHSIT) 1993–1996 J Hum Hypertens 2001 15 11 793 798 10.1038/sj.jhh.1001268 11687924

[B21] Lin KC Lin HY Chou P Community based epidemiological study on hyperuricemia and gout in Kin-Hu, Kinmen J Rheumatol 2000 27 4 1045 1050 10782835

[B22] Pan WH Lee MS Chuang SY Lin YC Fu ML Obesity pandemic, correlated factors and guidelines to define, screen and manage obesity in Taiwan Obes Rev 2008 9 Suppl 1 22 31 18307695 10.1111/j.1467-789X.2007.00434.x

[B23] Alberti KG Zimmet P Shaw J The metabolic syndrome–a new worldwide definition Lancet 2005 366 9491 1059 1062 10.1016/S0140-6736(05)67402-8 16182882

[B24] Schneider HJ Glaesmer H Klotsche J Bohler S Lehnert H Zeiher AM Accuracy of anthropometric indicators of obesity to predict cardiovascular risk J Clin Endocrinol Metab 2007 92 2 589 594 17105840 10.1210/jc.2006-0254

[B25] Schneider HJ Friedrich N Klotsche J Pieper L Nauck M John U The predictive value of different measures of obesity for incident cardiovascular events and mortality J Clin Endocrinol Metab 2010 95 4 1777 1785 10.1210/jc.2009-1584 20130075

[B26] Chu NF Wang DJ Liou SH Shieh SM Relationship between hyperuricemia and other cardiovascular disease risk factors among adult males in Taiwan Eur J Epidemiol 2000 16 1 13 17 10.1023/A:1007654507054 10780337

[B27] Villegas R Xiang YB Cai Q Fazio S Linton M Li H Prevalence and determinants of hyperuricemia in middle-aged, urban Chinese men Metab Syndr Relat Disord 2010 8 3 263 270 10.1089/met.2009.0084 20158446 PMC3136729

[B28] Chen CC Wang WS Chang HY Liu JS Chen YJ Heterogeneity of body mass index, waist circumference, and waist-to-hip ratio in predicting obesity-related metabolic disorders for Taiwanese aged 35–64 y Clin Nutr 2009 28 5 543 548 10.1016/j.clnu.2009.04.017 19473734

[B29] Chen JH Pan WH Hsu CC Yeh WT Chuang SY Chen PY Impact of obesity and hypertriglyceridemia on gout development with or without hyperuricemia: a prospective study Arthritis Care Res 2013 65 1 133 140 10.1002/acr.21824 22933424

[B30] Hsieh SD Yoshinaga H Muto T Waist-to-height ratio, a simple and practical index for assessing central fat distribution and metabolic risk in Japanese men and women Int J Obes Relat Metab Disord 2003 27 5 610 616 10.1038/sj.ijo.0802259 12704405

[B31] Lyu LC Hsu CY Yeh CY Lee MS Huang SH Chen CL A case–control study of the association of diet and obesity with gout in Taiwan Am J Clin Nutr 2003 78 4 690 701 14522726 10.1093/ajcn/78.4.690

[B32] Pluta RM Shmerling RH Burke AE Livingston EH JAMA patient page. Gout JAMA 2012 308 20 2161 10.1001/jama.2012.4095 23188039

[B33] McAdams MA Maynard JW Baer AN Kottgen A Clipp S Coresh J Reliability and sensitivity of the self-report of physician-diagnosed gout in the campaign against cancer and heart disease and the atherosclerosis risk in the community cohorts J Rheumatol 2011 38 1 135 141 10.3899/jrheum.100418 21123328 PMC3285109

